# Assessment of Socio-Demographic Factors, Mother and Child Health Status, Water, Sanitation, and Hygienic Conditions Existing in a Hilly Rural Village of Nepal

**DOI:** 10.3390/ijerph16203965

**Published:** 2019-10-17

**Authors:** Pratibha Bhandari, JeongEun Bak, Kang-Sung Lee, Yun Chon, Anuj Bhattachan, Pradip Rimal, Biswo Ram Shrestha, Binayak Bhandari, Jeong-Ook Moon, Namkyu Wu, Won-Shik Chu, Chul-Ki Song, Caroline S. Lee, Vittal Mogasale, Sung-Hoon Ahn

**Affiliations:** 1 Department of Nursing, College of Health & Welfare, Woosong University, Daejeon 300718, Korea; pratibha@wsu.ac.kr; 2 International Vaccine Institute, Seoul 08826, Korea; jeongeun.bak@gmail.com (J.B.); talktosung1@gmail.com (K.-S.L.); yunchon@gmail.com (Y.C.); ipdlah@gmail.com (A.B.); 3 Epidemiology and Disease Control Division, Department of Health Services, Teku, Kathmandu 44600, Nepal; pradiprimal2029@gmail.com; 4 District Public Health Office, Nuwakot 44900, Nepal; brstha2013@gmail.com; 5 Department of Railroad Integrated System Engineering, Woosong University, Daejeon 300718, Korea; binayak@sis.ac.kr; 6 Innovative Design and Integrated Manufacturing Nepal, Grishma Marg, Babarmahal-11, Kathmandu 44600, Nepal; 7 Department of Mechanical and Aerospace Engineering, Seoul National University, Seoul 08826, Korea; munjw777@snu.ac.kr (J.-O.M.); xcb0395@snu.ac.kr (N.W.); 8 Department of Mechanical Convergence Engineering, Gyeongsang National University, Changwon 51390, Korea; wschu@gnu.ac.kr; 9 Department of Mechanical Engineering, Gyeongsang National University, Jinju 52828, Korea; cksong@gnu.ac.kr; 10 Department of Materials and Chemical Engineering, Hanyang University, Ansan 15588, Korea; sunyonglee@hanyang.ac.kr

**Keywords:** socio-demographic, mother and child health, water, sanitation, hygiene, immunization, social network, Nepal

## Abstract

In many low income developing countries, socioeconomic, environmental and demographic factors have been linked to around half of the disease related deaths that occur each year. The aim of this study is to investigate the sociodemographic factors, mother and child health status, water, sanitation, and hygienic conditions of a Nepalese community residing in a hilly rural village, and to identify factors associated with mother and child health status and the occurrence of diarrheal and febrile disease. A community-based cross-sectional survey was carried out and 315 households from the village of Narjamandap were included in this study. Factors associated with diarrhea, febrile disease, and full maternal and under-five immunizations were assessed using logistic regression. Results showed that higher education level (middle school versus primary education; Odds Ratio (OR): 0.55, *p* = 0.04; high school versus primary education; OR 0.21, *p* = 0.001) and having a toilet facility at home were significantly associated with a lower risk of developing diarrhea and febrile disease (OR 0.49, *p* = 0.01), while, interestingly, the use of improved water supply was associated with higher risk (OR 3.07, *p* = 0.005). In terms of maternal immunization, the odds of receiving a tetanus toxoid vaccination were higher in women who had regular antenatal checkups (OR 12.9, *p* < 0.001), and in those who developed complications during pregnancy (OR 4.54, *p* = 0.04); for under-five immunization, the odds of receiving full vaccination were higher among children from households that reported diarrhea (OR 2.76, *p* < 0.001). The findings of this study indicated that gaps still exist in the mother and child healthcare being provided, in terms of receiving antenatal checkups and basic immunizations, as evidenced by irregular antenatal checkups, incomplete and zero vaccination cases, and higher under-five deaths. Specific public health interventions to promote maternal health and the health of under-five children are suggested.

## 1. Introduction

Around 52% of deaths in low-income countries in 2015 occurred because of pregnancy-related complications, childbirth, postpartum care, water and vector borne diseases, nutritional, and vaccine preventable diseases [1].

There are many factors linked to morbidity and mortality, such as socio-economic and demographic characteristics of the families, education status, geographical location, age of the mother, water, sanitation and hygiene facilities, and the health care delivery system [2,3]. Considerable amounts of resources, both within the country and in collaboration with international agencies, are being directed towards improving overall public health.

Nepal is a small, landlocked, developing country located between India and China. Nepal is naturally endowed with wide variety of landscapes, climates, and associated flora and fauna, and is inhabited by diverse groups of people with different languages and cultures. Despite this, its geographical location (being landlocked), difficult topography, and political instability are the main factors hindering health care reforms in the country [4]. As of 2016, around 80% of the total population lives in rural areas [5], where health care services are limited. In addition, many of the mountainous areas are sparsely populated hence provision of basic health care services, such as antenatal care, immunization, and institutionalized delivery, becomes more challenging. The number of health care workers available in the country is 7/10,000, below the standard 23/10,000 recommended by the World Health Organization [6].

Pregnant mothers and under-five children are most affected by these limitations. The under-five mortality rate in 2015 was 39/1000 for live births and the maternal mortality rate was 258/100,000 for live births [7]. The common causes of maternal mortality are hemorrhage, sepsis, unsafe abortions, and labor related complications [8]; for children under five years of age, common causes include respiratory infections, malnutrition, diarrheal diseases, and intrapartum related complications [9]. Even though there has been a remarkable decline in the maternal and under-five mortality ratios since the 1990s [3,10], disparities, depending on geographical areas [11], socio-economic status, educational background, and ethnicity, still exist [3]. In addition, it has been reported that delays in vaccination timings increase the risk of mortality [2,12].

**Figure 1:**
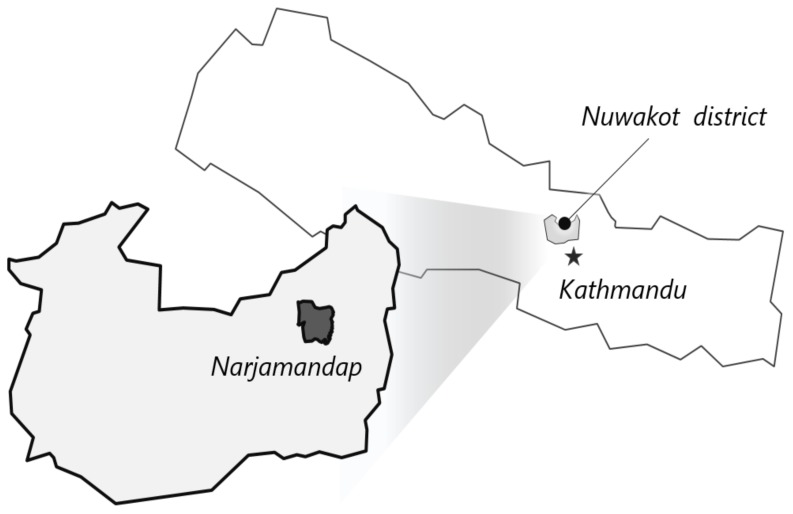
The Narjamandap community of the Nuwakot district where a vaccine delivery system was developed using renewable energy sources for remote areas of Nepal; the star in the figure indicates the capital of Nepal, Kathmandu.

In order to reduce these disparities and make timely health care available to all, several community-based, decentralized programs have been implemented throughout the country since the mid-1990s [10,11,13]. Several initiatives have been taken on by the Government of Nepal (GoN) to scale up the existing health care services in collaboration with international organizations. Additionally, many voluntary non-governmental organizations have contributed to social welfare, health promotion, and community development [14]. In accordance with the initiatives taken by the GoN, a mother and child health (MCH) survey was conducted on 20–21 December 2015 to identify and understand the gaps between the current health status and existing health targets of the Narjamandap Village Development Committee (VDC) of the Nuwakot District, Nepal (Figure 1). This project was undertaken by the Global Solar Volunteer Corps (GSVC) and was technically supported by the International Vaccine Institute (IVI). This survey was part of a larger project, the “Construction of Vaccine Delivery System in High Altitude Remote Area in Nepal using Renewable Energy”, funded by the National Research Foundation of Korea and aiming to increase the vaccination coverage rates in developing countries, especially in Nepal where geographic and environmental barriers such as high altitude and poor road conditions hinder community access to vaccines.

In Nepal in recent years, the use of smart phones has greatly increased and is being used as a means to ‘stay connected’ even when physical barriers may exist for actual contact. For example, during monsoons villages may be completely cut off due to heavy landslides but people may still be able to ‘stay connected’ through social networking sites. Previous studies reported a positive correlation between social networking site (SNS) interventions and health behavior changes [15]. Hence, to obtain baseline information, we also assessed the use of smart phones, internet, and, specifically, SNSs like Facebook in our study. This was done in order to plan for future technology-based health promotion.

The main aim of this study was to assess the basic demographic factors and the general health and immunization status of under-five children and mothers in the Narjamandap community of Nepal. We expect the findings of this survey to provide useful baseline information, identify gaps in the MCH care to formulate public health policies, and enhance MCH interventions in Narjamnadap Village and other low-income developing countries. 

The specific objectives are as follows:

- To describe the demographic and household characteristics in Narjamandap Village.
- To describe the mother and child health status particularly related to antenatal care and routine immunization in Narjamandap Village.
- To understand the water, sanitation and hygiene (WASH)status in Narjamandap Village.
- To identify factors associated with MCH status.
- To identify factors associated with the occurrence of diarrheal and febrile disease.
- To assess the usage of smart phones and SNSs by the local population.
- To generate recommendations to improve the existing MCH care in Nepal by identifying the gaps in system, delivery, and care.

## 2. Materials and Methods

### 2.1. Participants and Procedure

A community-based cross-sectional study was conducted. The convenience sampling method was used in this survey. All 365 households from a total of 9 wards in the Narjamandap community were targeted for the survey. The questionnaire was administered to all mothers having a child who was under-five years of age. If there were no children under five years of age residing in that household then another adult was selected as a respondent. On the other hand, if there was more than one mother with children under five years of age in the household then all mothers were included as separate respondents in the survey.

### 2.2. Measures

The researchers prepared the survey questionnaire based on the Nepal Demographic Health Survey, which included information on socio-demographic and household characteristics, MCH status, and water, sanitation and hygiene characteristics.

The operational definitions for acute diarrhea and fever adopted for our study were as follows: Acute diarrhea referred to the passage of three or more episodes of liquid or loose stool per day, lasting less than 14 days within last four weeks; any documented or undocumented rise in body temperature was considered to be the presence of fever. The mothers were asked whether they, their children, or anyone in their family had incidence of diarrhea or fever within the past four weeks.

The current use of and future intention to use smart phones and social networking sites was also assessed.

### 2.3. Data Collection

Data collection was done in December 2015. The survey was conducted by local surveyors who utilized two IVI monitors and were aided by female community health volunteers (FCHV) who spoke the Nepali language. The surveyors were trained for half a day by the researchers prior to the actual data collection. Permission to conduct the survey was given by the local and the district health offices. The purpose and methodology of the survey, nature of participation (voluntary), confidentiality issues and how the data would be used and transferred was explained to all the participants and written informed consent, as part of the survey form, was obtained from all participants.

### 2.4. Data Analysis

Data was analyzed using SAS Version 9.4. Descriptive statistics were used to analyze the socio-demographic characteristics. Multivariate stepwise logistic regression was performed to identify the factors associated with acute diarrhea and fever, full maternal immunization, and full vaccination in children under five years of age. Variables were considered significant at 0.15 or 0.2 and added to the model. The level of significance was set to 0.05. If the subject responded ‘Yes’ to both diarrhea and fever, it was recoded as ‘Yes’ for disease and included as dependent variable. The independent variables were occupation, education status, source of drinking water, and presence of a toilet.

## 3. Results

### 3.1. Socio-Demographic Characteristics

Of the total 365 mothers screened and enrolled for the study survey, 315 cases were included in this analysis, after exclusion of incomplete survey forms and data cleaning. The majority of the participants were 25–34 years old. Around 80% of the respondents were housewives engaged in farming. The most common form of ‘property’ possessed by the respondents were radio, television and land. (Refer to Table 1 for detailed sociodemographic information.)

### 3.2. Water, Sanitation, and Hygiene Characteristics

The prevalence of acute diarrhea and fever in the household over a four-week period was reported to be 21.6% and 35.2% respectively. Around 91.5% of the households reported access to improved water supply. The majority of the households (96.8%) reported no proper water treatment before use. Around 86.7% of the mothers reported washing their hands after visiting toilet and approximately 50% of them reported washing their hands before handling food. Detailed characteristics of water, sanitation, and hygiene are shown in Table 2.

### 3.3. Maternal and Child Health Characteristics

The mean age of marriage and first childbirth was 19 and 20 years, respectively. Around 8% of the mothers did not receive any antenatal checkups. The majority of the mothers (77%) had one experience of pregnancy, while 2% of them had been pregnant four to five times (irrespective of the outcome). Around 14% reported developing complications during pregnancy. The most common complications were fever with rash (8.4%) followed by vaginal bleeding (2.6%) and seizure (1.3%). Stillbirth as an outcome of pregnancy was reported by 1% of the mothers. The primary birth weight reported was between 2.5 and 3.5 kg (range: 1.8–5kg), of which around 95% were reported alive and well. (Table 3)

The total number of under-five children in our study was 464. Under-five death was reported for 2.6% of the live births. From the responses received, incomplete doses of OPV and DPT-Hib-HepB were reported for two children and zero dose vaccinations for measles and rubella (MR) and Japanese encephalitis (JE) was reported for one child. The reported reason for the incomplete doses was unwillingness to vaccinate the child further, and for the zero dose was lack of awareness about vaccination.

### 3.4. Factors Associated with Acute Diarrhea and Fever

The result of the logistic regression analysis showed that mothers who had secondary level education were 44% less likely to develop disease (*p* = 0.04, 95% Confidence interval (CI) 0.31–0.9) than mothers who had only primary education. Similarly, the odds of mothers with high school education developing a disease were 79% lower compared to mothers with only primary education (*p* = 0.001, 95% CI = 0.08–0.56). In terms of occupation, being a shopkeeper was associated with a 73% lower risk of developing a disease. The results showed that the odds of developing a disease were 3.07 times higher among the families who used improved sources of drinking water, whereas the odds were 50% lower for families who had toilets in their house. The results are displayed in Table 4.

### 3.5. Factors Associated with Full Antenatal and Under-Five Child Immunization

Tetanus toxoid (TT) vaccination status was included as a dependent variable whereas antenatal care (regular/irregular), complications during pregnancy, place of delivery and use of smart phone were included as independent variables. The odds of receiving TT vaccination increased with regular antenatal checkups Odds ratio (OR) 12.9, 95% CI 4.77–35.26, *p* < 0.001), developing any complications during pregnancy (OR 4.54, 95% CI 1.02–20.08, *p* = 0.04), and the use of smart phones (OR 4.98, 95% CI 1.39–17.84, *p* = 0.01).

In this study, full vaccination in an under-five child referred to a dose of BCG, three doses of OPV and the pentavalent vaccine (DPT-Hib-HepB), and one dose of the measles and rubella vaccine. The odds of under-five children receiving full vaccination were higher in families who reported diarrhea within the 4 weeks before the survey (OR 2.76, 95% CI 1.57–4.85, *p* < 0.001), and among families who had a Facebook account (OR 2.55, 95% CI 1.40–4.63, *p* = 0.002). We also assessed the correlation between self-reported BCG vaccination, presence of BCG scar, and immunization card during the survey. We considered those who had an immunization card and reported the date of vaccination as ‘truly vaccinated’ children. In the truly vaccinated group, 90.1% of the respondents self-reported BCG vaccination and 80.1% of them were found to have a BCG scar.

## 4. Discussion

This study investigated the factors associated with maternal and child health in a rural hilly village of Nepal located about 55 kilometers from the capital city Kathmandu. The prevalence, within a four-week period, of acute diarrhea and fever among the study population during the survey (December–January) was reported to be 21.6% and 35.2% respectively, which was high compared to the findings of the Nepal Demographic and Health Survey (NDHS) [3]. The NDHS reported acute diarrhea and fever within the two weeks before the survey as 8% and 21%, respectively, specifically for under-five children; however, our study assessed acute diarrhea and fever in any member of the family within the past 4 weeks. In our study, 2.6% of under-five deaths were reported from the live births. In developing countries, diarrhea and febrile disease have been linked to high under-five mortality. Although the specific cause of under-five deaths was not explored in our study, our findings require further exploration and attention. Identification of the cause will help to plan targeted interventions to reduce under-five deaths. The survey for our study was conducted between the months of December and January, which are the coldest and driest months when there is water scarcity. The higher prevalence of diarrhea and fever could be related to unsanitary and unhygienic practices during these months.

Consistent with the NDHS results, around 91.5% of the households had access to improved sources of drinking water, however, only 10% of them were treating the water before drinking. The commonly reported water treatment measures were boiling or adding chlorine. In our study, around 60.6% of the households had a toilet facility in their house, which is similar to the report from the NDHS 2016 (62%). Handwashing with soap and water was a practice reported by the majority of the mothers, mostly after defecation and before eating. Although handwashing has been recognized as a simple yet effective means of prevention against many diseases, in many places, including Nepal, it is practiced only before eating and after defecation [16,17]. Public education campaigns targeting the need for handwashing after handling body secretions or before handling food, etc., will greatly benefit this community. Additionally, follow up reinforcement and demonstration by the grass root level health workers is required in order to facilitate translation of this knowledge into everyday practice [18].

Nepal, as a country, has made significant progress in reducing the maternal morbidity rate by promoting regular antenatal checkups, institution delivery, etc. According to the NDHS 2016, 83.5% of mothers in this region received regular antenatal care; our findings were similar to the NDHS (79.4%; 95% CI 0.74–0.83). The community where the survey was conducted has nine wards, of which Ward 9 has a higher population but is more geographically isolated. Since 80% of the area in Nepal is rural, with the majority residing in difficult geographical locations, use of innovative ways to provide health care for this type of population will further ensure maximum perinatal care coverage.

Fever with rash during the antenatal period was reported by 8.4% of the mothers in our study. Rubella infection could be one cause. Since the rubella vaccination was only introduced to the National Immunization Program in the year 2013, special projects and campaigns to vaccinate children (under 15 years) who have missed the previous supplemental immunization activities must be planned using innovative methods. In addition, our results showed that the odds of being fully vaccinated with the TT vaccine were significantly higher in mothers who had regular antenatal care, developed any complications during pregnancy and delivered in the hospital; similarly, the odds of receiving full vaccination for under-five children were significantly higher in children who had diarrhea—in other words, contact with health providers at any point during pregnancy, or the appearance of disease symptoms, significantly increased the chances of being vaccinated. In Nepal, each ward has a female community health volunteer (FCHV) from the local community who play a vital role in promoting the mother and child’s health. However, several challenges, relating to their roles and performance, still exist, like limitations on reporting and documenting due to remoteness, limited literacy status, and overburdening from multiple roles [19,20], and must be addressed. In addition, professional channels linking the grass root level to the higher level must be strengthened, whereby limitations of the grass root level workers can be addressed. Special monitoring and follow-up must be done to promote mother and child health within the populations residing in geographically isolated regions. Using technology-based, advanced interventions for surveillance and monitoring may prove useful. Based on our study results, it can be implied that self-reporting is a good indicator of true vaccination and it may be used as an indicator for true vaccination status in the absence of documentation, especially for BCG vaccination.

Consistent with the literature [21,22,23], the odds of having a disease (diarrhea and fever) were significantly reduced in mothers who had secondary or high school level education and for those who had a toilet facility. Interestingly, in our study, being a shopkeeper was associated with lower risk of developing a disease. In contrast with our result, in a study based in Ethiopia, Mihrete et al. (2014) reported that having a ‘non-agricultural’ job was related with higher risk of developing diarrhea [24]. The shopkeepers in our study were mostly small-scale tea-shop/restaurant owners or retailers. The positive association with lower risk for disease may be due to better sanitation or hygienic practices because poor hygienic conditions may affect customer turnover. In addition, these places also commonly turn on the radio or television as a means of customer entertainment; hence, it could be that awareness of prevention and transmission is higher within this group of people.

In our study, contrary to general expectations, use of improved sources of drinking water was associated with higher risk of developing a disease. Specifically, use of piped water was associated with higher risk of disease, but the result was not significant for the public tap. In the case of Nepal, depending on the geographical location of the wards in the village, the source of water supply can be different. Normally, the water from the source is collected and treated in an underground reservoir tank by the government and is supplied to the village at certain times of the day through public taps. In rural areas, since the houses are sparsely distributed, the water from the public tap is further extended, temporarily, to houses or localities which are further from the area where the public tap is installed. In almost all cases, the extended distribution system is not a closed system and is at the surface level where there is a high chance of breakage and contamination, especially during the monsoon season. This finding is a matter of public health concern. Strong measures must be taken to improve the water distribution channels. In addition, people must be educated about the need for water treatment, demonstration of ways to treat drinking water, and safe collection and storage of drinking water.

In addition to the general maternal and child health status, we also explored the use of technology among the households. Specifically, we were interested to know about the use of smart phones and social networking sites. Since the use of social media is escalating, we were interested to obtain baseline data that could help us in planning future technology-based interventions to promote mother and child health. Interestingly, our results showed that the odds for receiving full vaccination, both for the under-five children and pregnant mothers, were significantly higher for families who used Facebook. In the case of families who owned a smart phone, the odds of receiving TT vaccine was five times higher. While the mechanism for this is not fully clear, we posit that the use of smart phones and access to social networking sites might lead to higher awareness and health care seeking behavior. Future studies can be directed to explore this further. Recently, the use of smart phones has accelerated in many areas of Nepal, including the rural areas. Even in the absence of proper electricity supply, houses use small-scale renewable sources to generate electricity, which is being used mainly for lighting and charging mobile phones [25]. The use of social networking sites as a possible tool for interacting with the community, monitoring health status and promoting health must be explored in the future.

While this study comprehensively assessed many important factors relating to the health of the community, some limitations existed. The information collected in relation to antenatal care and under-five immunization, prevalence of diarrhea and fever, etc., was subjective information based on the mother’s recall; the mothers may not have known whether other adults in the family had any episodes of diarrhea recently. In addition, the longer recall period for incidence of diarrhea (four weeks) used in this study may have affected the findings. Since this was a cross-sectional survey, causation cannot be attributed to the independent variables. As the survey was conducted in a remote rural area, generalizing the findings, for rest of Nepal, should be done with caution.

## 5. Conclusions

Although most reported literatures have described the factors associated with MCH status or WASH conditions in rural Nepal, this study comprehensively assessed the household and sociodemographic characteristics and their relation with MCH and WASH conditions in a semi-urban area of Nepal. The findings from this study highlighted some gaps that still exist in the mother and child health care being provided, in terms of receiving antenatal checkups, basic immunization as evidenced by irregular antenatal checkups, incomplete and zero vaccination cases, and higher under-five deaths. In addition, the use of improved source of drinking water was associated with higher risk of developing acute diarrhea and febrile disease. A positive association between the use of smart phones, Facebook and receiving full immunization was also found in our study. Public health implications that can be derived from this study are as follows:

- The existing health care delivery system must be strengthened to reach all communities in a timely manner.
- Ways to minimize the time gap between the discovery of symptoms, reporting, and action must be clearly planned. This could be in done economically, technologically, via infrastructure (e.g., use of drones to provide medical supplies for areas that are geographically isolated, or using telemedicine to guide actions, online data entry and disseminating services) or travelling facilities for local level workers.
- In addition, professional health care monitoring teams (experts) must be deployed promptly to further investigate any cases of complications during pregnancy, under-five deaths, missed vaccinations, etc. These must be reported and appropriate actions must be taken promptly.
- Health care delivery for people living in geographically difficult terrains must be strengthened.
- In areas like our study setting, where there is an advantage of having uninterrupted power supply most of the time, the government must allocate resources and personnel for training and using technology to document, report, and monitor the health status of the community.
- Innovative use of mobile technology and social networking sites must be explored to promote the health of the community.

## Figures and Tables

**Table 1:** Sociodemographic characteristics (*N* = 315).

Variables	*N*	*n* (%)	Mean (SD)
Age			25.44 (5.93)
Ethnic group	301		
Dalit		22 (7.3)	
Tamang/Lama/Gurung		171 (56.8)	
Brahmin/Chettri		105 (34.9)	
Newar		3 (1)	
Occupation	315		
Housewife		70 (22.2)	
Farmer		47 (14.9)	
Shopkeeper		3 (1.0)	
Teacher		3 (1.0)	
Tailor		2 (0.6)	
Housewife & part time income generating worker *		187 ((59.7)	
Farmer & other part time work		3 ( (0.9)	
			
Ability to read & write	308		
Yes		239 (77.6)	
No		69 (22.4)	
Highest level of education	256		
Informal education		50 (19.5)	
Primary school (Grade 1–5)		62 (24.2)	

Middle school (Grades 6–8)		55 (21.5)	

Secondary (Grades 9 & 10)		51 (19.9)	

High school(Grades 11 & 12)		38 (14.8)	

			
Fuel used for cooking	287		
Firewood		240 (83.6)	
Biogas		1 (0.3)	
Firewood & other sources (kerosene, biogas, electricity)		46 (16)	
Daily source of lighting	293		
Electricity		260 (88.7)	
Kerosene		2 (0.7)	
Electricity & kerosene/candle		31 (10.6)	

* Other part time income generating works included farming, shop/restaurant owner, tailor and teacher.

**Table 2:** Water, sanitation and hygiene characteristics (*N* = 315).

Variables	*N*	*n* (%)
Sick with acute diarrhea in past 4 weeks	315	
Yes		68 (21.6)
No		247 (78.4)
Sick with fever in past 4 weeks	315	
Yes		111 (35.2)
No		204 ( 64.8)
Main source of drinking water *	293	
Improved sources		268 (91.5)
Unimproved sources		25 (8.5)
		
Water treatment	315	
Yes		10 (3.2)
No		305 (96.8)
Type of water treatment	10	
Boil		6 (60)
Add chlorine		2 (20)
Water filter		2 (20)
Toilet facility	315	
Yes		191 (60.6)
No		124 (39.4)
Type of toilet	187	
Flush toilet with public sewerage		30 (16)
Flush toilet with septic tank		76 (406)
Outdoor pit latrine		81 (43.3)
Hand washing practice before handling food	293	
Soap & water		159 (54.3)
Water only		102 (34.8)
Mud/Ash/Sand		1 (0.3)
Both Soap & water & water only		18 (6.1)
Water only & mud/ash/sand		6 (2.0)
Soap & water, water only & mud/ash/sand		1 (0.3)
		
Hand washing practice before eating	293	
Soap & water		175 (59.7)
Water only		101 (34.5)
Mud/ash/sand		2 (0.7)
Soap & water & water only		9 (3.1)
Soap & water & mud/ash/sand		6 (2.0)
		
Hand washing practice after visiting toilet	293	
Soap & water		254 (86.7)
Water only		11 (3.8)
Mud/ash/sand		12 (4.1)
Soap & water & water only		7 (2.4)
Soap & water & mud/ash/sand		9 (3.1)

* Improved sources: piped water into dwelling/yard/plot, public tap, tube well, protected dug well and spring, rainwater; unimproved sources: unprotected dug well, spring and surface water, tanker and bottled water.

**Table 3:** Maternal and child health characteristics (*N* = 315).

Variables	*N*	*n* (%)	Mean (SD)
Age at marriage	278		19.04 (3.45)
Age at first childbirth	278		20.74 (3.44)
Total surviving children	293		1.58 (1.28)
TT vaccine for recent pregnancy	315		
Yes		257 (81.6)	
No		58 (18.4)	
TT vaccine dose	254		
One dose		3 (1.2)	
Two doses		251 (98.8)	
Missing data		61	
Antenatal care during pregnancy	315		
Regular		250 (79.4)	
Less than three visits		39 (12.4)	
None		26 (8.3)	
Type of delivery			
Normal vaginal delivery	315	298 (94.6)	
Assisted delivery (vacuum cup/forceps)		1 (0.3)	
Caesarean section		16 (5.1)	
Place of delivery	315		
Home		164 (52.1)	
Health post		66 (21.0)	
Private clinic		7 (2.2)	
Hospital		78 (24.8)	
Immunization card	290		
Yes		137 (47.2)	
No		153 (52.8)	
			
BCG	206		
Yes		199 (96.6)	
No		7 (3.4)	
			
BCG scar	277		
Yes		233 (84.1)	
No		44 (15.1)	
			
Place of immunization	230		
Health post		133 (57.8)	
Booth		93 (40.4)	
Private clinic		4 (1.7)	

* TT: Tetanus toxoid; BCG: Bacille Calmette Guerin.

**Table 4:** Factors associated with acute diarrhea and fever.

Variables	Odd Ratio Estimate	95% Wald Confidence Limits	*P* Value
Education Primary education	1		
Illiterate	0.53	0.27–1.06	0.07
Secondary Education	0.55	0.31–0.97	0.04
High School	0.21	0.08–0.56	0.001
Housewife	1.67	0.87–3.20	0.12
Shopkeeper	0.27	0.08–0.83	0.02
Source of drinking water Improved Vs. Unimproved	3.07	1.38–6.81	0.005
Toilet facility	0.49	0.28–85	0.01
